# Immunohistochemical scoring of LAG-3 in conjunction with CD8 in the tumor microenvironment predicts response to immunotherapy in hepatocellular carcinoma

**DOI:** 10.3389/fimmu.2023.1150985

**Published:** 2023-06-05

**Authors:** Chun Chau Lawrence Cheung, Yong Hock Justin Seah, Juntao Fang, Nicole Hyacinth Calpatura Orpilla, Mai Chan Lau, Chun Jye Lim, Xinru Lim, Justina Nadia Li Wen Lee, Jeffrey Chun Tatt Lim, Sherlly Lim, Qing Cheng, Han Chong Toh, Su Pin Choo, Suat Ying Lee, Joycelyn Jie Xin Lee, Jin Liu, Tony Kiat Hon Lim, David Tai, Joe Yeong

**Affiliations:** ^1^ Department of Anatomical Pathology, Singapore General Hospital, Singapore, Singapore; ^2^ Duke-NUS Medical School, Singapore, Singapore; ^3^ Yong Loo Lin School of Medicine, National University of Singapore, Singapore, Singapore; ^4^ Temasek Polytechnic, Singapore, Singapore; ^5^ Institute of Molecular and Cell Biology (IMCB), Agency of Science, Technology, and Research (A*STAR), Singapore, Singapore; ^6^ Center of Statistical Research, School of Statistics, Southwestern University of Finance and Economics, Chengdu, Sichuan, China; ^7^ Division of Medical Oncology, National Cancer Centre Singapore, Singapore, Singapore; ^8^ Singapore Immunology Network (SIgN), Agency of Science, Technology, and Research (A*STAR), Singapore, Singapore

**Keywords:** LAG-3, CD8, immunotherapy, immune checkpoint blockade, biomarkers, hepatocellular carcinoma, multiplex immunohistochemistry, immunohistochemistry

## Abstract

**Introduction:**

Immune checkpoint blockade (ICB) is a systemic therapeutic option for advanced hepatocellular carcinoma (HCC). However, low patient response rates necessitate the development of robust predictive biomarkers that identify individuals who will benefit from ICB. A 4-gene inflammatory signature, comprising *CD8*, *PD-L1*, *LAG-3*, and *STAT1*, was recently shown to be associated with a better overall response to ICB in various cancer types. Here, we examined whether tissue protein expression of CD8, PD-L1, LAG-3, and STAT1 predicts response to ICB in HCC.

**Methods:**

HCC samples from 191 Asian patients, comprising resection specimens from 124 patients (ICB-naïve) and pre-treatment specimens from 67 advanced HCC patients treated with ICB (ICB-treated), were analyzed for CD8, PD-L1, LAG-3, and STAT1 tissue expression using multiplex immunohistochemistry followed by statistical and survival analyses.

**Results:**

Immunohistochemical and survival analyses of ICB-naïve samples showed that high LAG-3 expression was associated with shorter median progression-free survival (mPFS) and overall survival (mOS). Analysis of ICB-treated samples revealed that high proportions of LAG-3^+^ and LAG-3^+^CD8^+^ cells pre-treatment were most closely associated with longer mPFS and mOS. Using a log-likelihood model, adding the total LAG-3^+^ cell proportion to the total CD8^+^ cell proportion significantly increased the predictive values for mPFS and mOS, compared with the total CD8^+^ cell proportion alone. Moreover, levels of CD8 and STAT1, but not PD-L1, were significantly correlated with better responses to ICB. After analyzing viral-related and non-viral HCC samples separately, only the LAG3^+^CD8^+^ cell proportion was significantly associated with responses to ICB regardless of viral status.

**Conclusion:**

Immunohistochemical scoring of pre-treatment levels of LAG-3 and CD8 in the tumor microenvironment may help predict ICB benefits in HCC patients. Furthermore, immunohistochemistry-based techniques offer the advantage of being readily translatable in the clinical setting.

## Introduction

Hepatocellular carcinoma (HCC) is the sixth most common cancer and the third leading cause of cancer-associated mortalities globally (1). Many factors increase an individual’s risk of HCC, and the risk varies across geographical locations. Hepatitis B (HBV) and aflatoxin exposure are major risk factors in sub-Saharan Africa and eastern Asia, whereas hepatitis C, metabolic diseases, and alcoholism are primary risk factors in the USA and Europe. Treatment options for advanced HCC are often limited to systemic therapies, and the median survival is between 6 and 20 months (2).

Immune-checkpoint blockade (ICB) has shown encouraging efficacy in the treatment of HCC (3, 4). The Phase III CheckMate 459 clinical trial demonstrated that nivolumab was associated with clinically meaningful improvements in overall survival (OS), objective response rate, and complete response rate compared to sorafenib, although the primary endpoint OS did not reach the level of statistical significance (5). In addition, nivolumab is associated with fewer treatment-related adverse events and a higher therapy compliance rate. However, response rates to anti-programmed cell death protein 1/anti-programmed death-ligand 1 (anti-PD-1/anti-PD-L1) ICB remain suboptimal, with only a subset of patients benefitting from ICB monotherapy (6). While associated with higher response rates and survival, atezolizumab-bevacizumab combination therapy is also associated with higher treatment-related toxicities and financial costs (7). A biomarker-directed therapeutic strategy that maximizes treatment benefits and minimizes toxicities is clearly needed.

There are, however, no widely recognized blood or tissue biomarkers for predicting HCC response to ICB therapies in clinical use, and studies on serum biomarkers, such as alpha-fetoprotein (AFP), have returned inconsistent results (7–9). Peripheral immune cell profiling, cytokines, circulating tumor DNA and cells, tumor mutational burden, microsatellite instability, and gut microbiota have been evaluated as potential predictive markers with variable results (10–16). The identification of predictive RNA and protein biomarkers in the tumor microenvironment using techniques such as RNA-sequencing (RNA-seq) and immunohistochemistry (IHC) has become a major focus of research interest (17–19). The main advantage of IHC-based methods over RNA assays is that they are more readily translatable to, and adoptable in, clinical practice. The tissue expression of programmed death-ligand 1 (PD-L1) has been extensively explored by IHC for use as a predictive biomarker in HCC, although studies have yielded inconclusive results (5, 20, 21). The reliability of tissue PD-L1 status alone as a predictive biomarker is affected by the inter-assay heterogeneity of tissue PD-L1 expression when different IHC assay platforms are employed (22), and by issues of tumor heterogeneity and intra-observer variability (23). This evidence suggests that a single biomarker may not provide the best predictive value, and the identification of alternative tissue biomarkers is required.

Previously, our group found that IHC scoring of CD38^+^ and CD38^+^CD68^+^ cell densities in the tumor microenvironment predicted HCC patient responses to anti-PD-1/anti-PD-L1 ICB (24). Another promising predictive marker for HCC is the 4-gene inflammatory signature consisting of the following genes: cluster of differentiation 8 (*CD8*), *PD-L1*, lymphocyte-activating gene 3 (*LAG-3*), and signal transducer and activator of transcription 1 (*STAT1*) genes (17). Various trials demonstrated that upregulated expression of the 4-gene inflammatory signature is associated with response to immunotherapy in several cancers, including HCC, melanoma, and gastroesophageal cancer (17, 25, 26). In the HCC CheckMate 040 clinical trial, expression of 4-gene inflammatory signature, as determined by RNA-seq, was associated with an improved response to nivolumab and better OS (17).

In this study, the pre-treatment protein expression levels of CD8, PD-L1, LAG-3, and STAT-1 proteins within the HCC tumor microenvironment were determined using multiplex immunohistochemistry/immunofluorescence (mIHC/IF). We then determined whether the expression levels of the biomarkers were associated with overall response rates (ORR), progression-free survival (PFS), and overall survival (OS). We propose that pre-treatment levels of LAG-3^+^ and CD8^+^ cells in tumor tissue should be explored further to help identify HCC patients likely to benefit from immunotherapy using IHC-based techniques that are readily accessible during routine clinical care.

## Materials and methods

### Patients and tumors

Formalin-fixed paraffin-embedded (FFPE) HCC tissues and peripheral blood mononuclear cells (PBMCs) from 191 Asian patients with advanced HCC were obtained from the Department of Anatomical Pathology, Division of Pathology, Singapore General Hospital. A total of 124 patients who underwent tumor resection and had never received ICB treatment between March 1997 and July 2007 (ICB-naïve cohort), and 67 patients who received ICB treatment between May 2016 and March 2021 (ICB-treated cohort), were included. ICB-treated cohort samples were obtained prior to the initiation of ICB treatment. The clinicopathological parameters of the ICB-naïve and ICB-treated cohorts are summarized in **Supplementary Tables 1** and **2**, respectively.

HCC tumors were staged according to the AJCC or BCLC staging systems and graded according to the Edmondson–Steiner grading system. Responses were determined according to RECIST V.1.1 (27). Patients who achieved a best response of complete response or partial response according to RECIST V.1.1 were termed responders and patients who achieved a best response of stable disease or progressive disease according to RECIST 1.1 were termed non-responders. The Centralized Institutional Review Board of SingHealth provided ethical approval for the use of patient materials in this study (CIRB Ref: 2009/907/B).

### Multiplex immunohistochemistry/immunofluorescence analysis

A total of 191 FFPE tissue sections (4-μm thick) from the aforementioned patients were first stained with hematoxylin (Leica Biosystems Richmond Inc., Richmond, IL, USA) and eosin (Merck KGaA, Darmstadt, Germany), and three representative areas of high cellularity were chosen for mIHC/IF staining, which was performed using an Opal Multiplex Fluorescence IHC kit (Akoya Biosciences, Marlborough, MA, USA), as previously described (24, 28–30). In brief, tissue sections were first incubated with primary antibodies against PD-L1, LAG-3, CD8, CD38, CD68, and STAT1 (**Supplementary Table 3**), followed by polymeric horseradish peroxidase-conjugated secondary antibodies (Leica Biosystems Inc.); appropriate positive and negative controls were included. Opal fluorophore-conjugated tyramide signal-amplification (TSA) buffer (Akoya Biosciences) was then added and after the heat-stable deposition of the TSA-conjugated fluorophore around the marker of interest, the slides were subjected to heat-induced epitope retrieval. The process was repeated until all markers were labeled, at which point spectral DAPI (Akoya Biosciences) was added. Fluorescence images were captured using a Vectra 3.0 pathology imaging system microscope (Akoya Biosciences) and analyzed using inForm Cell Analysis Software (Akoya Biosciences) and HALO (Indica Labs, Albuquerque, NM, USA).

The densities of PD-L1^+^, LAG-3^+^, CD8^+^, CD38^+^, CD68^+^, and STAT1^+^ cells in the tumor microenvironment were determined as cell counts per a pre-defined, high-powered field (334 µm x 250 µm). The cell proportions of the biomarkers were determined by normalization with DAPI using the following formula:


$$\text{Cell proportion}=\frac{{\text{Number of biomarker}}^{+}\text{ cells}}{{\text{Number of DAPI}}^{+}\text{ cells }}\;\times \;100\%$$

Samples were then categorized as ‘high’ or ‘low’ according to whether the cell proportion was above or below the cut-off points (best thresholds) that produced the lowest *P*-value determined using Determine the Optimal Cutpoint for Continuous Variables method in R 4.1.1 (24, 29, 30).

### Single-cell RNA sequencing

PBMCs from 6 ICB-treated patients were extracted for single-cell RNA sequencing. Approximately 16,000 PBMCs were loaded onto the Chromium Controller (10× Genomics, San Francisco, CA, USA) for targeted recovery of 10,000 single cells. The cells were partitioned into nanoliter-scale Gel Bead-In Emulsions and individually barcoded. The 10× Genomics Chromium Single Cell 3′ Reagent Kit v3 (10× Genomics, San Francisco, CA, USA) was used for reverse transcription, cDNA amplification, and library construction of gene expression libraries according to the manufacturer’s instructions. Library quality was assessed using BioAnalyzer 2100 with an Agilent High Sensitivity DNA Kit (Agilent Technologies, Inc., Santa Clara, CA, USA). Paired-end sequencing at 2 × 150-bp was performed using the Illumina NovaSeq 6000 platform (Illumina, Inc., San Diego, CA, USA). The raw reads were aligned and quantified using the Cell Ranger (version 4.0.0, 10× Genomics) against the GRCh38 human reference genome (GenBank Assembly ID GCA_000001405.28). The gene expression data were cleaned up and processed using Seurat R package (v4.0.3) with the default parameters unless otherwise specified, as follows: (i) excluding cells with<200 unique genes (low-quality cells or empty droplets), >4,000 unique genes (doublets), or >35% mitochondrial genes (low-quality or dying cells), and subsequently (ii) normalization (SCTransform), (iii) projection to lower dimensional space (RunPCA and RunUMAP), (iv) cell clustering (FindNeighbors and FindClusters), and (v) mapping to references of 2,700 PBMC cells with cell-type annotation (FindTransferAnchors, MapQuery) (31).

### Flow cytometry

PBMCs from 4 ICB-treated patients were incubated with Zombie NIR Fixable Viability dye (BioLegend, San Diego, CA, USA) for 10 min at 4°C in the dark for live/dead cell discrimination. Fc receptors were blocked with Human TruStain FcX (BioLegend) for 10 min at room temperature. Cell surfaces were labelled with antibodies targeting markers of interest (**Supplementary Table 4**) for 30 min at 4°C. Sample data were acquired on a Cytek Aurora spectral flow cytometer (Cytek Biosciences, Fremont, CA, USA) and analyzed using FlowJo V.10 software (FlowJo LLC, Ashland, OR, USA) with the FlowJo plug-in to generate the uniform manifold approximation and projection plots.

### Validation, follow-up, and statistical analysis

Long-term follow-up data for patients were obtained from the medical records. Disease-free survival (DFS) was defined as the time from tumor resection to disease relapse. PFS was defined as the time from the start of treatment to disease progression. OS was defined as the time from the start of treatment to death or censoring at the date of the last follow-up. Median DFS (mDFS) was defined as the time at which 50% of the patients relapsed after tumor resection. Median PFS (mPFS) was defined as the time at which the disease had progressed in 50% of the patients. Median OS (mOS) was defined as the time at which 50% of the patients had died. Cox proportional hazards regression was performed to evaluate the effects of biomarker expression and clinicopathological parameters on PFS and OS. Multivariate Cox proportional hazards regression analysis of survival outcomes was performed while adjusting for AFP level, Eastern Cooperative Oncology Group Performance Status (ECOG PS) scale, macrovascular invasion status, and Child-Pugh score. Statistical analysis was conducted using R studio 2021.09.0 running R 4.1.1 (R-core Team, R Foundation for Statistical Computing, Vienna, Austria), and a *P*-value of<0.05 was considered to indicate a statistically significant difference.

## Results

### LAG-3 is a marker of poor prognosis in ICB-naïve resected HCC, but indicates a good prognosis in ICB-treated advanced HCC

**Figure 1:**
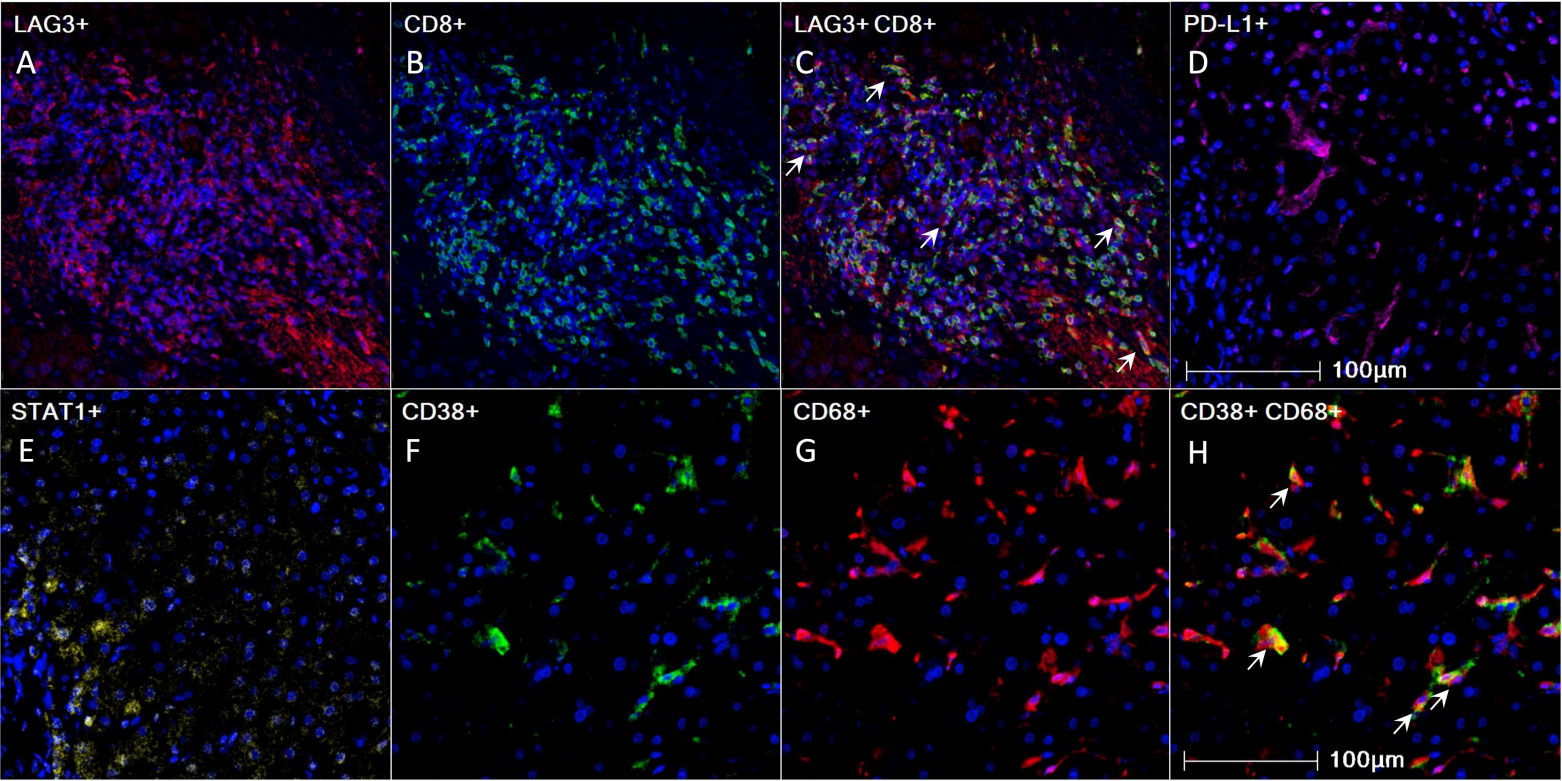
Pre-treatment 4-gene inflammatory signature, CD38, and CD68 expression in HCC tumor microenvironment as visualized using mIHC/IF. **(A-H)** Representative mIHC/IF images: **(A)** LAG-3 (red); **(B)** CD8 (green); **(C)** Colocalization of LAG-3 (red) and CD8 (green) in some cells, as indicated by white arrows; **(D)** PD-L1 (magenta); **(E)** STAT1 (yellow); **(F)** CD38 (green); **(G)** CD68 (red); **(H)** Colocalization of CD38 (green) and CD68 (red) in some cells, as indicated by white arrows. DAPI was stained blue. Scale bar: 100 µm. HCC, hepatocellular carcinoma; mIHC/IF, multiplex immunohistochemistry/immunofluorescence.

**Figure 2:**
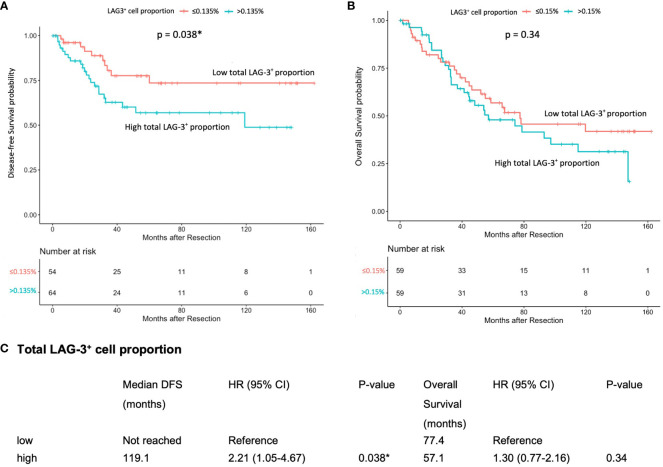
Association between pre-treatment total LAG-3^+^ cell proportion and post-resection DFS or OS in ICB-naïve patients (n = 124). **(A)** Kaplan-Meier curve illustrating a significant association between high total LAG-3^+^ cell proportion and shorter mDFS after resection (HR = 2.21, *P* = 0.038). **(B)** Kaplan-Meier curve illustrating an insignificant association between high total LAG-3^+^ cell proportion and shorter mOS after resection (HR = 1.30, *P* = 0.34). **(C)** Univariate Cox regression analysis of total LAG-3^+^ cell proportion of ICB-naïve patients using optimal cut-off point. CI, confidence internal; mDFS, median disease-free survival; mOS, median overall survival; HR, hazard ratio; ICB, immune checkpoint blockade. *P-value< 0.05 indicates statistical significance.

**Figure 3:**
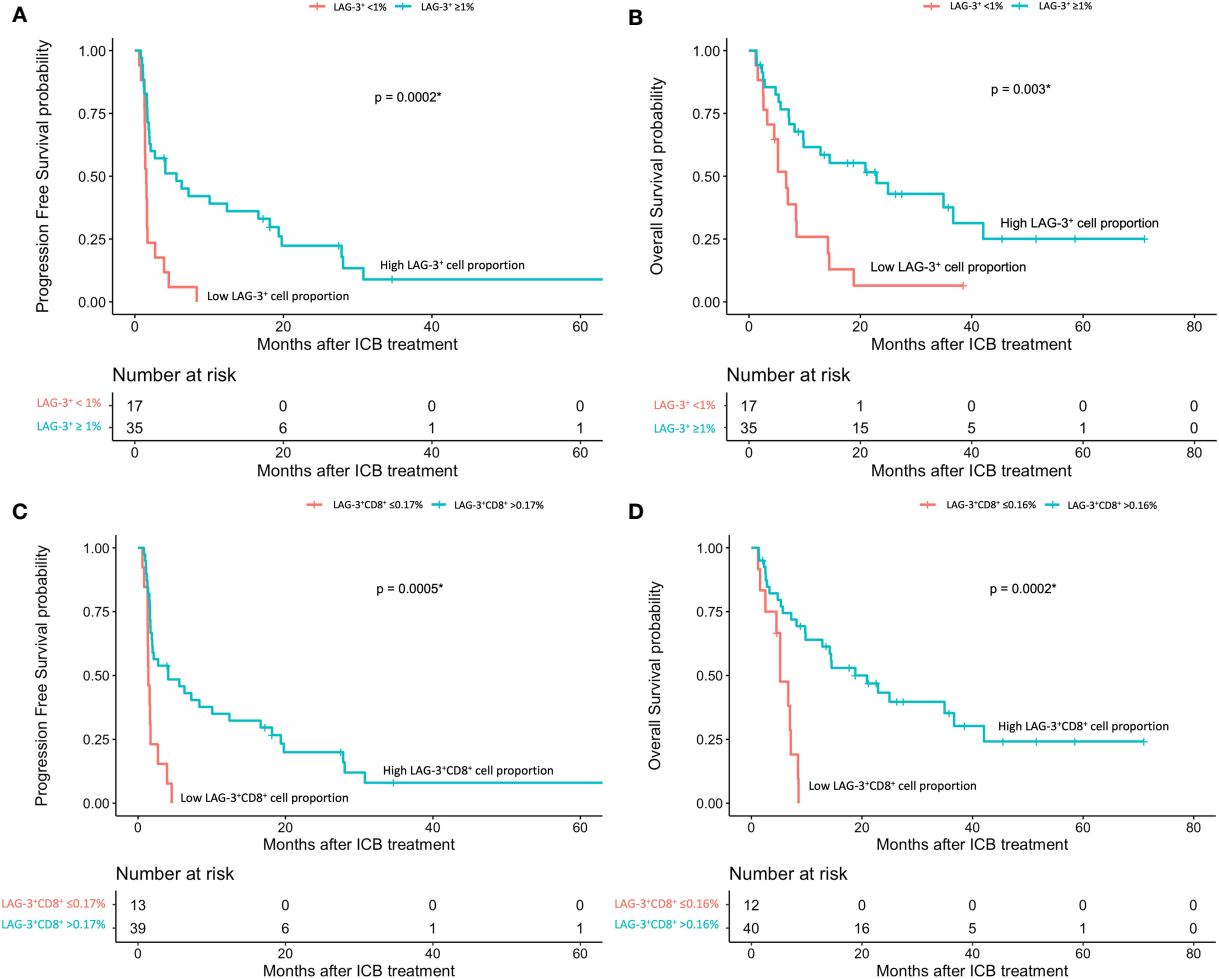
HCC patients’ response to ICB in relation to pre-treatment total LAG-3^+^ and LAG-3^+^CD8^+^ cell proportions (n = 67). **(A, B)** Kaplan-Meier curve showing the association between high LAG-3^+^ cell proportion and better progression-free survival (mPFS: high 5.6 months vs low 1.53 months, HR = 0.278, *P*< 0.001) **(A)** or overall survival (mOS: high 22.9 months vs low 6.7 months, HR = 0.350, *P* = 0.003) **(B)** after treatment with ICB. **(C, D)** Kaplan-Meier curve showing the association between high total LAG-3^+^CD8^+^ cell proportion and better progression-free survival (mPFS: high 4.1 months vs low 1.4 months, HR = 0.276, *P*< 0.001) **(C)** or overall survival (mOS: high 20.9 months vs low 5.2 months, HR = 0.200, *P*< 0.001) **(D)** after treatment with ICB. HCC, hepatocellular carcinoma; HR, hazard ratio; ICB, immune checkpoint blockade; mPFS, median progression-free survival; mOS, median overall survival. *P-value< 0.05 indicates statistical significance.

The mIHC/IF analysis of HCC tissue samples confirmed the expression of LAG-3 within the tumor microenvironment (**Figure 1A**). Compared with patients who had lower LAG-3 expression, Cox regression analysis demonstrated that ICB-naïve patients with upregulated tissue LAG-3 expression had significantly shorter mDFS (119.1 months vs mDFS not reached, *P* = 0.038, HR = 2.21; **Figures 2A, C**) and shorter, but not statistically significant, mOS (57.1 months vs 77.4 months, *P* = 0.34, HR = 1.30; **Figures 2B, C**). In contrast, univariate Cox regression analysis of tissue LAG-3 expression in ICB-treated patients using an optimal cut-off of 1% demonstrated that patients with a high pre-treatment total LAG-3^+^ cell proportion had a longer mPFS (5.6 months) and mOS (22.9 months), compared with 1.5 and 6.7 months, respectively, for patients with a low total LAG-3^+^ cell proportion (mPFS: p< 0.001, HR = 0.278; mOS: *P* = 0.003, HR = 0.350; **Table 1**, **Figures 3A, B**). Among the various clinicopathological parameters, only the Child-Pugh score significantly predicted mOS in our ICB-treated cohort (**Table 2**). Multivariate analysis confirmed the association between a high total LAG-3^+^ cell proportion and better mPFS and mOS in ICB-treated patients after adjusting for clinical prognostic factors, including AFP level, ECOG PS scale, macrovascular invasion status, and Child-Pugh score (**Table 1**, **Supplementary Table 5**).

**Table 1:** Univariate and multivariate Cox proportional hazards regression analysis of median PFS (mPFS) and median OS (mOS) in patients with HCC treated with ICB (n = 67).

	Progression-free survival					Overall survival					
	Univariate analysis			Multivariate analysis		Univariate analysis			Multivariate analysis		
Variable/ cell proportion	mPFS (months)	HR (95% CI)	*P*-value	HR (95% CI)	*P*-value	mOS (months)	HR (95% CI)	*P*-value	HR (95% CI)	*P*-value	
LAG-3											
Low	1.53	Reference		Reference		6.67	Reference		Reference	
High	5.60	0.278 (0.141-0.55)	0.0002*	0.224 (0.106-0.47)	0.00008*	22.90	0.350 (0.175-0.70)	0.003*	0.307 (0.147-0.64)	0.002*
LAG-3^+^CD8^+^											
Low	1.40	Reference		Reference		5.20	Reference		Reference	
High	4.10	0.276 (0.135-0.57)	0.0005*	0.259 (0.123-0.55)	0.0004*	20.90	0.200 (0.085-0.47)	0.0002*	0.208 (0.088-0.49)	0.0004*
CD8											
Low	1.93	Reference		Reference		4.87	Reference		Reference	
High	12.40	0.46 (0.24-0.89)	0.021*	0.280 (0.127-0.61)	0.002*	14.40	0.182 (0.068-0.49)	0.0007*	0.160 (0.057-0.45)	0.0005*
PD-L1											
Low	1.77	Reference		Reference		9.80	Reference		Reference	
High	2.70	0.66 (0.380-1.12)	0.13	0.56 (0.317-0.98)	0.044*	18.80	0.73 (0.41-1.30)	0.28	0.58 (0.306-1.10)	0.09
STAT1											
Low	1.57	Reference		Reference		6.67	Reference		Reference	
High	4.10	0.380 (0.176-0.82)	0.014*	0.34 (0.136-0.87)	0.024*	22.90	0.314 (0.136-0.73)	0.007*	0.276 (0.104-0.73)	0.010*
CD38											
Low	1.77	Reference		Reference		8.17	Reference		Reference	
High	11.24	0.49 (0.266-0.89)	0.02*	0.44 (0.235-0.83)	0.011*	36.67	0.267 (0.122-0.58)	0.0009*	0.216 (0.095-0.49)	0.0003*
CD38^+^CD68^+^											
Low	7.97	Reference		Reference		7.97	Reference		Reference	
High	19.2	0.51 (0.300-0.87)	0.014*	0.51 (0.293-0.88)	0.015*	34.93	0.381 (0.207-0.70)	0.002*	0.354 (0.186-0.67)	0.002*

• *P-value< 0.05 indicates statistical significance.

• In multivariate Cox regression analysis, survival outcome was adjusted for AFP level, ECOG PS scale, macrovascular invasion status, and Child-Pugh score. Full analysis is shown in **Supplementary Table 4**.

• AFP, alpha-fetoprotein; CI, confidence interval; ECOG PS, Eastern Cooperative Oncology Group score Performance Status; HCC, hepatocellular carcinoma; HR, hazard ratio; ICB, immune checkpoint blockade; mPFS, median progression-free survival; mOS, median overall survival.

**Table 2:** Univariate Cox regression analysis of median overall survival (mOS) in ICB-treated HCC patients (n = 67).

Patient factor	mOS (months)	Hazard ratio (95% CI)	*P*-value
Hepatitis status			
Negative	12.8	Reference	
Positive	14.5	1.07 (0.58-1.98)	0.84
BCLC Stage			
A and B	20.9	Reference	
C	14.2	0.87 (0.375-2.10)	0.87
Edmondson–Steiner Grade			
1	12.8	Reference	
2 and 3	8.4	0.84 (0.396-1.79)	0.65
Age (years)			
<65	9.7	Reference	
≥65	14.5	1.20 (0.66-2.18)	0.55
AFP marker (ng/mL)			
<400	14.2	Reference	
≥400	19.2	0.97 (0.53-1.79)	0.93
ECOG PS scale			
0	14.5	Reference	
≥1	7.0	0.75 (0.40-1.40)	0.37
Child-Pugh score			
A5	19.3	Reference	
A6	5.7	0.71 (0.365-1.37)	0.30
B7 and B8	4.1	0.387 (0.151-0.99)	0.047*
Macrovascular invasion			
Absent	14.7	Reference	
Present	9.8	1.01 (0.53-1.93)	0.97
Extra-hepatic Spread			
Absent	8.0	Reference	
Present	14.7	1.43 (0.76-2.70)	0.27

• *P-value< 0.05 indicates statistical significance.

• AFP, alpha-fetoprotein; BCLC, Barcelona Clinic Liver Cancer; CI, confidence interval; ECOG PS, Eastern Cooperative Oncology Group Performance Status; HCC, hepatocellular carcinoma; HR, hazard ratio; ICB, immune checkpoint blockade; mOS, median overall survival.

### Pre-treatment LAG-3^+^CD8^+^, CD8^+^, STAT1^+^, and CD38^+^ cells were also associated with better prognosis in ICB-treated HCC

**Figure 4:**
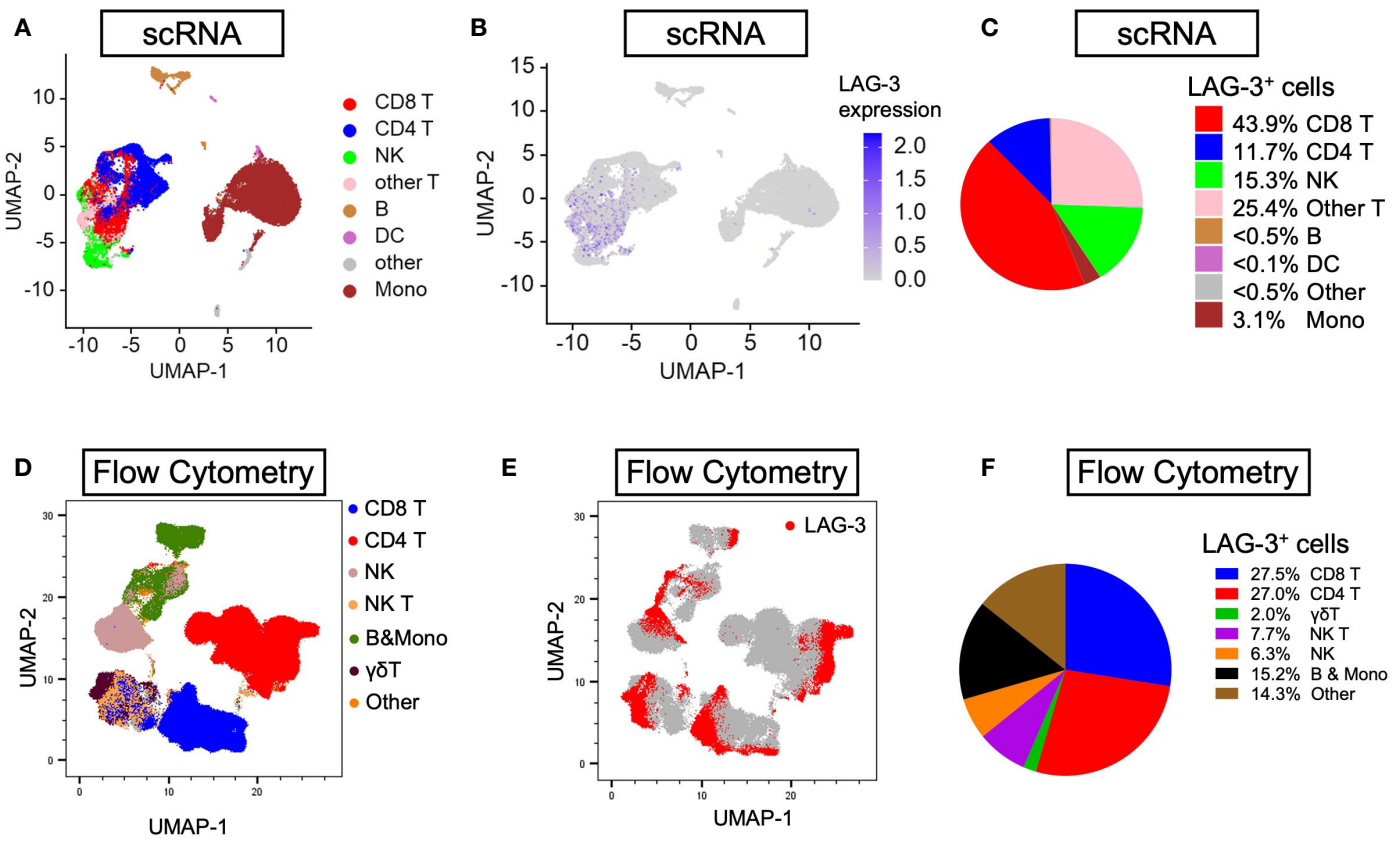
Distribution of LAG-3 expression by lineages of PBMCs isolated from HCC patients. **(A, B)** Uniform manifold approximation and projection (UMAP) plots illustrating abundance of **(A)** eight main lineages and **(B)** average LAG-3 gene expression from six PBMCs (three responders and three non-responders) investigated by scRNA-seq. **(C)** Pie chart showing frequency of LAG-3-expressing cells by individual lineages. **(D, E)** UMAP plots depicting abundance of **(D)** seven main lineages and **(E)** LAG-3 protein expression of four HCC PBMCs (two responders and two non-responders) studied by flow cytometry. **(F)** Pie chart showing frequency of LAG-3-expressing cells for each cell type. B, B cells; DC, dendritic cells; HCC, hepatocellular carcinoma; LAMP-2, lysosome-associated membrane protein 2; Mono, monocytes; NK, natural killer cells; NK T, natural killer T cells; UMAP, uniform manifold approximation and projection.

Next, to examine and characterize the immune cell types that expressed LAG-3, we performed both single-cell RNA sequencing (scRNA-seq) and flow cytometry analysis of PBMCs from the HCC patients. As shown in **Figure 4**, LAG-3 was predominantly expressed by CD8^+^ T cells (43.9% by scRNA-seq and 27.5% by flow cytometry). Therefore, we further investigated whether the LAG3^+^CD8^+^ T cell proportion correlated with survival outcomes of ICB-treated HCC patients. Similar to patients with a high LAG-3^+^ cell proportion, ICB-treated patients with a high total LAG-3^+^CD8^+^ cell proportion had significantly longer mPFS (4.1 months vs 1.4 months, p< 0.001, HR = 0.276; **Table 1**, **Figure 3C**) and mOS (20.9 months vs 5.2 months, p< 0.001, HR = 0.200; **Table 1**, **Figure 3D**), and the association remained significant after adjusting for clinical prognostic factors (**Table 1**, **Supplementary Table 5**). Overall, the total LAG-3^+^CD8^+^ cell proportion appeared to be as good as, if not better than, the LAG-3^+^ cell proportion as a predictive marker for responses to ICB in HCC.

In addition, we investigated whether CD8, STAT1, PD-L1, CD38, and CD68 predict the survival outcomes of ICB-treated HCC patients. The expression of these markers within the tumor microenvironment was confirmed by mIHC/IF (**Figures 1B–H**). We found that high pre-treatment CD8^+^ and STAT1^+^ cell proportions were significantly associated with longer mPFS and mOS in ICB-treated patients, whereas PD-L1 expression showed an insignificant association (**Table 1**, **Supplementary Figure 1**). Patients with a high total CD8^+^ cell proportion had longer mPFS (12.4 months vs 1.9 months, *P* = 0.021, HR = 0.46; **Table 1**, **Supplementary Figure 1A**) and mOS (14.4 months vs 4.9 months, *P* < 0.001, HR = 0.182; **Table 1**, **Supplementary Figure 1B**). Similarly, patients with a high total STAT1^+^ cell proportion had longer mPFS (4.1 months vs 1.6 months, *P* = 0.014, HR = 0.38; **Table 1**, **Supplementary Figure 1C**) and mOS (22.9 months vs 6.7 months, *P* = 0.007, HR = 0.31; **Table 1**, **Supplementary Figure 1D**). Previously, our group showed that CD38^+^ and CD38^+^CD68^+^ cell densities predicted the responsiveness of HCC patients to immunotherapy (24). In accordance with the previous study, we found that patients with high CD38^+^ and CD38^+^CD68^+^ cell proportions also had significantly longer mPFS and mOS (**Table 1**, **Supplementary Figures 1E-H**).

**Figure 5:**
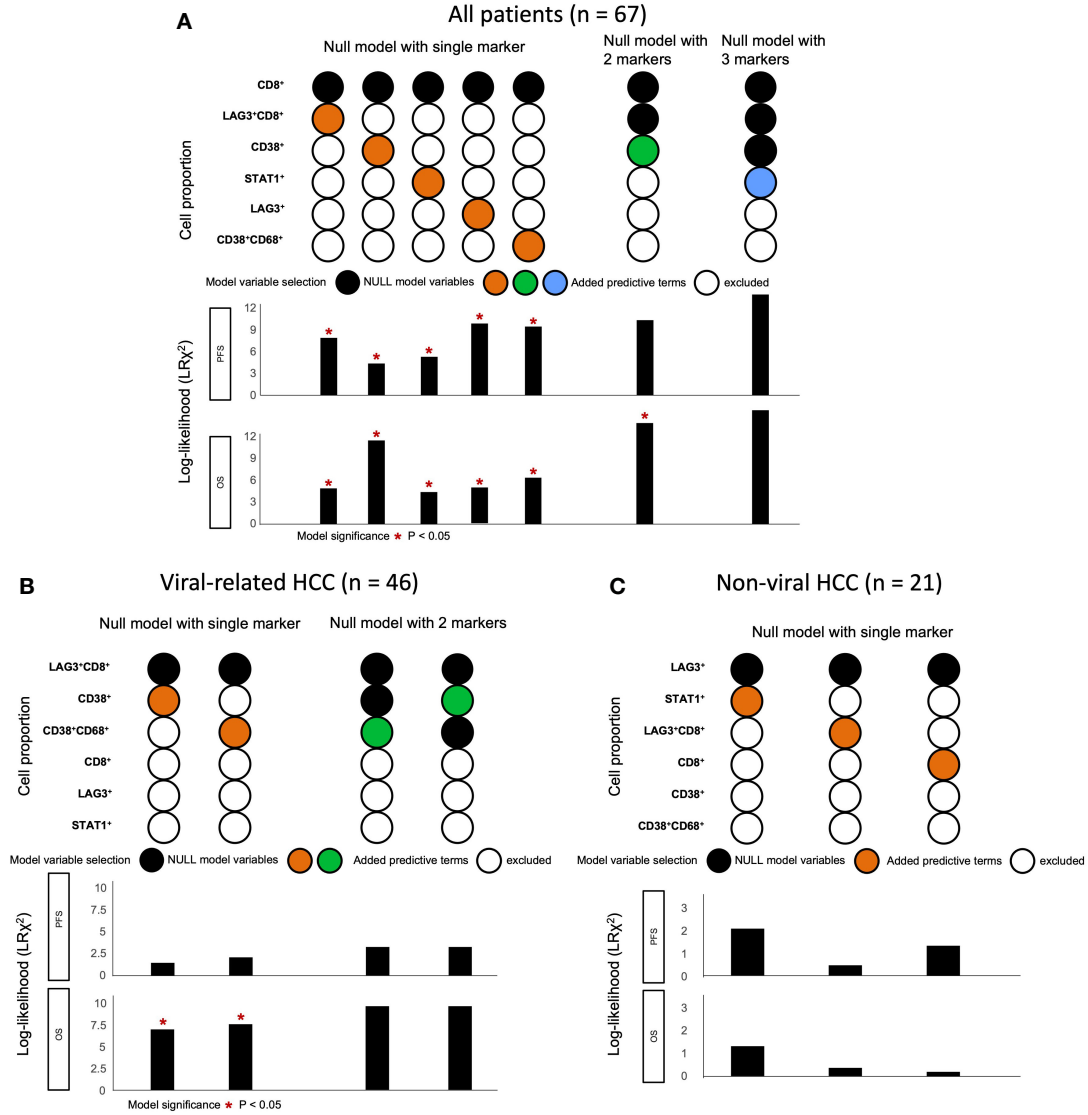
Change in log-likelihood of models with addition of predictive terms in ICB-treated cohort. **(A)** Log-likelihood models with predictive terms added for all ICB-treated HCC patients (n = 67). CD8^+^ cell proportion was used as basis for comparison, as it had the best hazard ratio for OS in multivariate analysis. Subsequent predictive terms were added and arranged according to increasing hazard ratio. The best models were selected and included in the figure from **Supplementary Table 6**. **(B)** Log-likelihood models with predictive terms added for ICB-treated patients with viral-related HCC (n = 46). LAG-3^+^CD8^+^ cell proportion was used as basis for comparison, as it had the best hazard ratio for OS in multivariate analysis. Subsequent predictive terms were added and arranged according to increasing hazard ratio. The best models were selected and included in the figure from **Supplementary Table 9**. **(C)** Log-likelihood models with predictive terms added for ICB-treated patients with non-viral HCC (n = 21). LAG-3^+^ cell proportion was used as the basis for comparison, as it had the best hazard ratio for OS in multivariate analysis. Subsequent predictive terms were added and arranged according to increasing hazard ratio. The best models were selected and included in the figure from **Supplementary Table 10**. Null model with two markers was not performed, as none of the single markers were significant. **(A-C)** *P-value< 0.05 indicated statistical significance, as determined with likelihood ratio test. HCC, hepatocellular carcinoma; ICB, immune checkpoint blockade; OS, overall survival.

Lastly, we used a log-likelihood model to ascertain whether multiple biomarkers are better predictors of survival outcomes than single biomarkers. As the CD8^+^ cell proportion provided the best hazard ratio for mOS in the multivariate analysis (**Table 1**), we used this cell proportion as the basis for comparison, and we added subsequent predictive terms based on the next best hazard ratios. We found that adding the total LAG-3^+^ cell proportion to the CD8^+^ cell proportion significantly enhanced the predictive value of the CD8^+^ cell proportion alone for both PFS and OS (PFS: ΔLRχ^2^ = 9.87, *P* = 0.002; OS: ΔLRχ^2^ = 4.92, *P* = 0.027; **Figure 5**, **Supplementary Table 6**), compared to CD8^+^ cell proportion alone. Similarly, adding the total LAG-3^+^CD8^+^ cell proportion to the CD8^+^ cell proportion significantly increased the predictive value of the CD8^+^ cell proportion alone for both PFS and OS (PFS: ΔLRχ^2^ = 7.9; *P* = 0.005; OS: ΔLRχ^2^ = 4.9; *P* = 0.0269; **Figure 5**, **Supplementary Table 6**), compared to CD8^+^ cell proportion alone.

### LAG-3^+^CD8^+^ expression was significantly associated with responses to ICB regardless of viral hepatitis status

**Figure 6:**
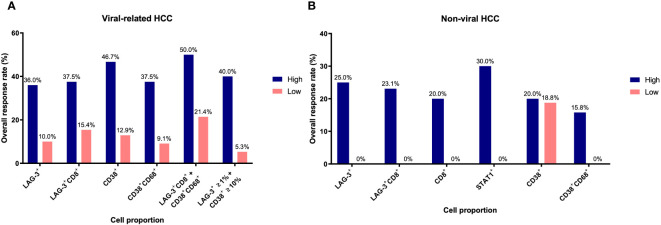
Overall response rates of biomarkers for overall survival based on viral status. **(A)** Overall response rates of LAG-3^+^, LAG-3^+^CD8^+^, CD38^+^, CD38^+^CD68^+^, LAG-3^+^CD8^+^ + CD38^+^CD68^+^, and LAG3^+^ ≥1% + CD38^+^ ≥10% cell proportions for overall survival in viral-related HCC (n = 46). **(B)** Overall response rates of LAG-3^+^, LAG-3^+^CD8^+^, CD8^+^, STAT1^+^, CD38^+^, and CD38^+^CD68^+^ cell proportions for overall survival in non-viral HCC (n = 21). Only those groups significant in univariate analysis (**Table 3**) are shown here. The receiver operating characteristic curves of each marker are shown in **Supplementary Figure 2**. HCC, hepatocellular carcinoma.

In our previous study, we found that CD38^+^ and CD38^+^CD68^+^ cell densities were significantly associated with responses to ICB in viral-related HCC but not non-viral HCC patients (24). To ascertain whether the same association existed in this cohort, we analyzed viral-related and non-viral HCC cases separately. Only the LAG-3^+^CD8^+^ cell proportion was found to be significantly associated with for OS regardless of viral status (**Table 3**, **Supplementary Table 7, 8**). Not surprisingly, patients with high LAG-3^+^CD8^+^ cell proportions had the best ORRs, with an ORR of 37.5% for viral-related HCC and 23.1% for non-viral HCC (**Figure 6**). On the other hand, the LAG-3^+^, CD8^+^, and STAT1^+^ cell proportions were significanly associated with ORR only with non-viral HCC, while the CD38^+^ and CD38^+^CD68^+^ cell proportions were significantly associated with ORR only with viral-related HCC.

**Table 3:** Univariate cox regression for overall survival of patients with viral-related (n = 46) and non-viral (n = 21) HCC treated with ICB.

Biomarker/ cell proportion	Viral-related HCC				Non-viral HCC			
	Univariate analysis		Multivariate analysis		Univariate analysis		Multivariate analysis	
	HR (95% CI)	*P*-value	HR (95% CI)	*P*-value	HR (95% CI)	*P*-value	HR (95% CI)	*P*-value
*LAG3*	0.41 (0.162-1.06)	0.066	–	–	0.038 (0.004-0.354)	0.004*	0.009 (0.0004-0.183)	0.002*
*LAG3^+^CD8^+^*	0.208 (0.048-0.91)	0.037*	0.022 (0.003-0.184)	0.0004*	0.092 (0.016-0.54)	0.008*	0.046 (0.004-0.53)	0.014*
*CD8*	0.149 (0.019-1.16)	0.069	–	–	0.197 (0.050-0.79)	0.021*	0.180 (0.033-0.98)	0.048*
*PD-L1*	0.65 (0.340-1.25)	0.20	–	–	1.08 (0.377-3.09)	0.89	–	–
*STAT1*	0.271 (0.035-2.10)	0.21	–	–	0.116 (0.022-0.61)	0.011*	0.041 (0.003-0.67)	0.025*
*CD38*	0.184 (0.063-0.54)	0.002*	0.158 (0.052-0.48)	0.001*	0.395 (0.108-1.44)	0.16	–	–
*CD68*	0.49 (0.222-1.07)	0.074	–	–	3.72 (0.49-28.4)	0.30	–	–
*CD38^+^CD68^+^*	0.251 (0.111-0.57)	0.0009*	0.251 (0.109-0.58)	0.001*	3.16^-10^ (0-Inf)	0.998	–	–

• *P-value< 0.05 indicates statistical significance.

• Multivariate analysis was performed only in cases where the univariate analysis was significant. Detailed analysis is shown in **Supplementary Table 7**.

• CI, confidence internal; HCC, hepatocellular carcinoma; HR, hazard ratio; ICB, immune checkpoint blockade.

**Figure 7:**
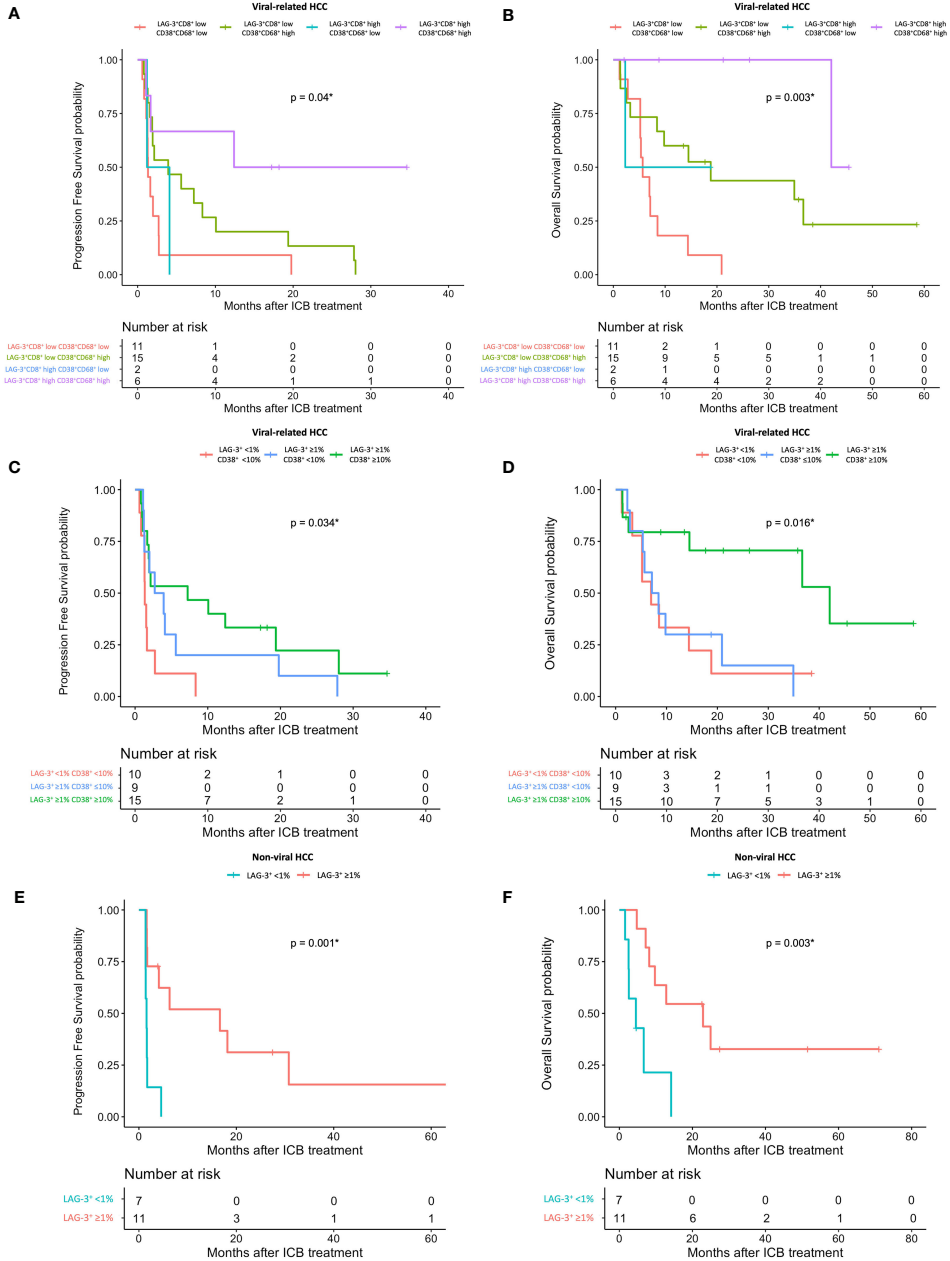
HCC patient’s response to ICB in relation to pre-treatment total LAG-3^+^ and LAG-3^+^CD8^+^ cell proportions depending on viral status. **(A, B)** Kaplan-Meier curve showing significant association between high LAG-3^+^CD8^+^ and CD38^+^CD68^+^ cell proportions and better mPFS **(A)** or mOS **(B)** after treatment with ICB in viral-related HCC (n = 46). **(C, D)** Kaplan-Meier curve showing significant association between high total LAG-3^+^ (≥1%) and CD38^+^ (≥10%) cell proportions and better mPFS **(C)** or mOS **(D)** after treatment with ICB in viral-related HCC (n = 46). **(E, F)** Kaplan-Meier curve showing significant association between high total LAG-3^+^ (≥1%) cell proportion and better mPFS **(E)** or mOS **(F)** after treatment with ICB in non-viral HCC (n = 21). HCC, hepatocellular carcinoma; ICB, immune checkpoint blockade, mPFS, median progression-free survival; mOS, median overall survival. *P-value< 0.05 indicates statistical significance.

Using the log-likelihood model, we found that, adding the CD38^+^CD68^+^ cell proportion to the LAG3^+^CD8^+^ cell proportion provided the best predictive value for OS for viral-related HCC compared to the LAG3^+^CD8^+^ cell proportion alone (**Δ**LRχ^2^ = 7.54, *P* = 0.006; **Figure 5**, **Supplementary Table 9**). Furthermore, viral-related HCC patients with high LAG3^+^CD8^+^ and CD38^+^CD68^+^ cell proportions had significantly longer mPFS and mOS, with an ORR reaching 50% (**Figures 6A**, **7A, B**). To establish a model that is more clinically appliable and easy to implement model by using just one biomarker, rather than two, we investigated the cell proportions in more detail and found that patients with ≥1% LAG3^+^ and ≥10% CD38^+^ cells had significantly longer mPFS and mOS, with an ORR of 40% (**Figures 6A**, **7C, D**), compared to patients with <1% LAG3^+^ and <10% CD38^+^ cells.

For non-viral HCC, none of the log-likelihood model adjustments to the LAG-3^+^ cell proportion were significant, indicating that this biomarker alone provided the best predictive value for OS (HR = 0.038, *P* = 0.004; **Table 3**) (**Supplementary Table 10**). Subsequent survival analysis demonstrated that non-viral HCC patients with a high LAG-3^+^ cell proportion had significantly longer mPFS and mOS, and an ORR of 25% (**Figures 6B**, **7E, F**).

To summarize, the best predictive models for PFS in ICB-treated patients involved adding the LAG-3^+^ cell proportion to the CD8^+^ cell proportion for all patients (**Δ**LRχ^2^ = 9.87, *P* = 0.002; **Figure 5**, **Supplementary Table 6**), adding the CD38^+^CD68^+^ cell proportion to the LAG-3^+^CD8^+^ cell proportion for viral-related HCC (**Δ**LRχ^2^ = 2.08, *P* = 0.15; **Figure 5**, **Supplementary Table 8**), and the LAG-3^+^ cell proportion alone for non-viral HCC (HR = 0.16, *P* = 0.004; **Supplementary Table 7**). The best predictive models for OS involved adding the CD38^+^ cell proportion to the CD8^+^ cell proportion for all patients (**Δ**LRχ^2^ = 11.5, *P* = 0.0004; **Figure 5**, **Supplementary Table 6**), adding the CD38^+^CD68^+^ cell proportion to the LAG-3^+^CD8^+^ cell proportion for virus-related HCC (**Δ**LRχ^2^ = 7.54, *P* = 0.006; **Figure 5**, **Supplementary Table 8**), and the LAG-3^+^ cell proportion alone for non-virus HCC (HR = 0.038, *P* = 0.004; **Table 3**).

## Discussion

In this study, we demonstrated the use of readily translatable mIHC/IF methods to determine the pre-treatment expression of the 4-gene inflammatory signature and CD38 expression in the HCC tumor microenvironment. Survival analysis established that high levels of cells expressing both LAG-3 and CD8 were most significantly associated with responses to ICB, regardless of viral status. LAG-3^+^, CD8^+^, and STAT1^+^ cell proportions also appeared to be associated with responses to ICB, although this depended on the viral status of the patients. Moreover, in accordance with our previous study (24), we found that high proportions of CD38^+^ cells, as well as CD38^+^CD68^+^ cells, were associated with improved responses to ICB, albeit only at a significant level in patients with viral hepatitis. Using a log-likelihood model, we demonstrated that adding the total LAG-3^+^ and LAG-3^+^CD8^+^ cell proportions to the total CD8^+^ cell proportion significantly increased the predictive values for both PFS and OS. Overall, the total LAG-3^+^ and LAG-3^+^CD8^+^ cell proportions appeared to be the best predictors of responses to ICB in patients with advanced HCC.

LAG-3 has been extensively evaluated for its potential as an immune-checkpoint target and predictive biomarker. Physiologically, the LAG-3 receptor acts as an inhibitory immune checkpoint and is expressed by activated T cells to prevent autoimmunity, autoinflammation, and tissue damage (32–35). In HCC, LAG-3 attenuates the effector function of CD8^+^ T cells, resulting in a less efficacious anti-tumoral response by the patient’s adaptive immune system (36). LAG-3 upregulation, which is observed in tumor-infiltrating lymphocytes in the majority of patients with HCC, is a mechanism of immune escape by tumors (37, 38). These findings implicate LAG-3 as a biomarker of poor prognosis in HCC. Indeed, it has been reported that high levels of LAG-3^+^ T cells are an independent predictor of poor PFS and OS in HCC patients (36), which is consistent with our analysis of the ICB-naïve cohort (**Figure 2**). Similar findings have been described for non-small cell lung cancer (39), head and neck squamous cell carcinoma (40), soft tissue sarcoma (41), melanoma (42), and renal cell carcinoma (43). Thus, it can be speculated that blocking the function of the LAG-3 receptor should reverse its immune checkpoint effect and restore the function of CD8^+^ T cells *via* a mechanism that is analogous to that underlying the effects of other widely used ICBs such as anti-PD-1 and anti-PD-L1. This hypothesis has prompted the development of the anti-LAG-3 antibody, relatlimab, which is undergoing extensive evaluation in numerous randomized trials of various cancer types, including HCC (44). A phase II/III clinical trial comparison of a combined relatlimab with nivolumab therapy versus nivolumab alone for melanoma showed an encouraging efficacy and safety profile for the combined therapy (45, 46), leading to the FDA approval of nivolumab and relatlimab as a combination therapy for advanced melanoma (47).

As ICBs are intended to stimulate an inhibited or exhausted anti-tumor immune response, it is logical to hypothesize that immune cells infiltrating the tumor-environment play an important role in responses to ICB, and that patients with a high density of such infiltrating immune cells would be more likely to respond to ICB. In accordance with this hypothesis, advanced melanoma patients with ≥1% tissue LAG-3 expression detected by IHC were shown to have longer median PFS after ICB treatment (48). Moreover, using the same cutoff of 1%, we found that ICB-treated HCC patients with high LAG-3^+^ cell proportions in the tumor microenvironment had significantly longer PFS and OS, compared to patients with low LAG-3^+^ cell proportions. Interestingly, flow cytometry analysis of pre-treatment peripheral blood from a cohort study of 188 ICB-treated melanoma patients and 94 ICB-treated urothelial cancer patients with the aim of identifying blood-based biomarkers showed that the presence of LAG-3^+^ and LAG-3^+^CD8^+^ T cells in peripheral blood was shown to be associated with poorer survival outcomes in ICB-treated melanoma and urothelial carcinoma (49). These conflicting findings may be due to differences in the nature and properties of peripheral immune cells and infiltrating immune cells, which represent two functionally distinct cell populations. Taken together, the data suggested that a pre-treatment tissue IHC LAG-3 expression cutoff of ≥1% may be clinically useful for evaluation of a patient’s likelihood of a positive response to ICB therapy. Furthermore, while high LAG-3 expression is a good predictive indicator of a better response to ICB, it is a marker of poor prognosis in HCC patients with no ICB treatment. These opposing findings suggest that the effects of LAG-3 on patient survival rate are unlikely to be due to its possible prognostic value. This also indicates that patients with high levels of LAG-3^+^ cells will benefit from ICB, as their prognosis would be very poor without any ICB treatment.

The independent high expression levels of two other immune markers, CD8 and STAT1, also demonstrated significant associations with survival outcomes in ICB-treated HCC patients. While the infiltration of CD8^+^ T cells into the tumor microenvironment showed a weak correlation with survival after anti-PD-1 treatment in the CheckMate 040 clinical trial (17), our study showed that high levels of CD8^+^ cells are also significantly associated with ICB response and may be used in conjunction with the LAG-3^+^ cell proportion to guide ICB treatment in clinical practice. On the other hand, the use of protein STAT1 levels as a predictive marker in HCC has not been reported. In the context of HCC, STAT1 is reportedly involved in the regulation of innate and adaptive immune responses within the tumor environment (50–55). An IHC human breast cancer study demonstrated that p-STAT1 is a potential marker for selecting patients for anti-PD-1/PD-L1 immunotherapy (56). Although our study demonstrated a relatively weak association between STAT1 and ICB responses, STAT1 expression in the HCC tumor microenvironment remains a potential predictive marker and warrants more in-depth evaluation. While PD-L1 status has been extensively evaluated as a predictive marker of ICB responsiveness, the results have been mixed and suboptimal at best. We did not identify clinically significant associations between PD-L1 expression and survival outcome in our previous and current ICB-treated patient cohorts (24); therefore, the clinical utility of PD-L1 remains to be seen.

Hepatitis B and C viruses play a key role in the pathogenesis of HCC and the definition of HCC-infiltrating immune cell phenotypes (57–59). Although little is known about the regulation of HBV-specific CD8^+^ T cell functions, studies have shown significantly higher LAG-3 expression levels in CD8^+^ T cells from patients with HBV (57). Previously, we reported that CD38 expression predicted responses to ICB in viral-related HCC but not non-viral HCC (24), which is consistent with the findings in this study (**Table 3**, **Supplementary Table 5**). This suggests that CD38 alone may not be clinically useful for predicting responses in patients with no history of viral hepatitis. After performing separate analyses of viral-related and non-viral HCC, we found that only the LAG-3^+^CD8^+^ cell proportion predicted responses to ICB regardless of the viral hepatitis status, with the overall best ORRs in both groups (**Figure 6**). An important finding of our current study was that viral-related and non-viral HCC have distinctive predictive biomarker profiles (**Table 3**). Our data suggest that combining the LAG-3^+^ or LAG3^+^CD8^+^ cell proportions with the CD38^+^ or CD38CD68^+^ cell proportions is more useful in patients with viral hepatitis, whereas the LAG-3^+^ and CD8^+^ cell proportions may be more useful in patients without viral hepatitis. Nevertheless, when the viral status is unknown, IHC staining for LAG-3 and CD8 provides three phenotypes that can still predict responses to ICB. Our best predictive models also suggested that different combinations of biomarkers may be used, depending on the patient’s viral hepatitis status (**Figure 5**).

The CheckMate 040 trial demonstrated that RNA levels of the 4-gene inflammatory signature, as determined by RNA-seq, were associated with an improved response to nivolumab therapy and better OS (17). However, to the best of our knowledge, this study is the first to demonstrate an association between the IHC-detected protein expression of LAG-3, CD8, and STAT1 and responsiveness to ICB in HCC patients. Our findings fill the translation gap between RNA-seq and clinical practice, as IHC is more accessible and less technically challenging than RNA-based analysis. We believe that LAG-3 and CD8 expression levels in the tumor microenvironment have potential value as predictive biomarkers of ICB responses in patients with advanced HCC prior to ICB treatment. We propose that pre-treatment LAG-3^+^ cell proportions, with a cutoff of 1%, may be used in conjunction with the CD8^+^ cell proportion to aid in the identification of patients who are likely to be better responders to ICB using readily translatable IHC-based methods available in the clinical setting. The limitations of this current study include the retrospective and heterogeneous nature of our patient cohorts, including the multiple types of immunotherapies they received. Another limitation is that tumour-infiltrating lymphocytes would be more ideal than PBMCs for studying the immune landscapes of LAG3^+^ cells, but we did not have access to tumour-infiltrating lymphocytes from the same patients. More studies, such as expression analyses with larger multi-national cohorts or randomized clinical trials, should be conducted to confirm our findings and to further evaluate the potential utility of the biomarkers.

## Conclusion

This study establishes an association between pre-treatment LAG-3, CD8, STAT1, and LAG-3^+^CD8^+^ tissue expression and responsiveness to monoclonal antibody immunotherapy in patients with advanced HCC, with the LAG-3^+^CD8^+^ cell proportion being the most favorable protein biomarker. In particular, we showed that IHC staining of LAG-3 and CD8, as both single markers and the double LAG-3^+^CD8^+^ phenotype, is useful for predicting responses to ICB in pre-treatment patients with advanced HCC. We further showed that the choice of markers may be guided by the patient’s viral hepatitis status, and IHC scoring of CD38 can be added to the biomarker panel if the patient has viral-related HCC. The tissue expression of the markers can be determined using readily available and translatable IHC-based techniques. Future investigations, such as expression analyses in a larger multinational cohort, should aim to test the validity of our current findings. Following validation in a larger independent cohort, we will strive to adopt these predictive biomarkers as routine screening modalities in clinical practice to facilitate the accurate identification of patients most suited to cancer immunotherapy in the current era of precision medicine.

## Data availability statement

The data presented in the study are deposited in the GEO repository, accession number GSE233405.

## Ethics statement

The studies involving human participants were reviewed and approved by Centralized Institutional Review Board of SingHealth (CIRB Ref: 2009/907/B). Written informed consent for participation was not required for this study in accordance with the national legislation and the institutional requirements.

## Author contributions

Conceptualization, design, and supervision: TL, DT, and JY. Data acquisition: HT, SC, SYL, JJL, and DT. Drafting of the manuscript: CC, YS, JF, NO, and JY. Assistance with histology-based techniques: JNL, JCL, and SL. Assistance with single-cell RNA sequencing and flow cytometry: ML, CL, and XL. Statistical analysis: QC and JL. All authors contributed to the article and approved the submitted version.
