# Supplementary material for: Immunohistochemical scoring of LAG-3 in conjunction with CD8 in the tumor microenvironment predicts response to immunotherapy in hepatocellular carcinoma
Source: Front Immunol. 2023 Jun 5;14:1150985. doi: 10.3389/fimmu.2023.1150985 (PMC10277502; doi:10.3389/fimmu.2023.1150985)
Supplement: Supplementary file 1 [file DataSheet_1.docx]

Supplementary Material

**Immunohistochemical scoring of LAG-3 in conjunction with CD8 in the tumor microenvironment predicts response to immunotherapy in hepatocellular carcinoma**

Chun Chau Lawrence Cheung, Yong Hock Justin Seah, Juntao Fang, Nicole Hyacinth Calpatura Orpilla, Mai Chan Lau, Chun Jye Lim, Xinru Lim, Justina Nadia Li Wen Lee, Jeffrey Chun Tatt Lim, Sherlly Lim, Qing Cheng, Han Chong Toh, Su Pin Choo, Suat Ying Lee, Joycelyn Jie Xin Lee, Jin Liu, Tony Kiat Hon Lim, David Tai, Joe Yeong^*^

**^*^Correspondence:** Dr. Joe Yeong, 61 Biopolis Drive Proteos, Singapore, 138673; yeongps@imcb.a-star.edu.sg; Telephone: +65 63214930; Twitter: @JoeYeong

**Supplementary Table 1.** Clinicopathological characteristics of patients in the ICB-naïve cohort (n = 124).

| **Factor** | **Frequency (proportion)** |
| --- | --- |
| **Age at surgery (years)** | |
| <65 | 70 (56.5%) |
| ≥65 | 54 (43.5%) |
| **Sex** |  |
| Male | 98 (79.0%) |
| Female | 26 (21.0%) |
| **Ethnicity** | |
| Chinese | 96 (77.4%) |
| Indian | 1 (0.8%) |
| Malay | 3 (2.4%) |
| Other | 24 (19.4%) |
| **Size of tumor (cm)** | |
| <20 | 122 (98.4%) |
| ≥20 | 2 (1.6%) |
| **Viral hepatitis status** | |
| Positive | 56 (43.5%) |
| Negative | 68 (56.5%) |
| **Histological grading of tumor (4-scale Edmondson and Steiner grading system)** | |
| 1 | 20 (16.1%) |
| 2 | 47 (37.9%) |
| 3 | 55 (44.4%) |
| NA | 2 (1.6%) |
| **Pathological stage (AJCC staging system)** | |
| I | 81 (65.3%) |
| II | 24 (19.4%) |
| III/IV | 18 (14.5%) |
| NA | 1 (0.8%) |

- Abbreviation: AJCC, American Joint Committee on Cancer; ICB, immune checkpoint blockade; NA, not applicable.

**`**

**Supplementary Table 2.** Clinicopathological characteristics of patients in the ICB-treated cohort (n = 67).

| Clinicopathological characteristics | Frequency (proportion) |
| --- | --- |
| **Age (years)** | |
| <65 | 27 (40.3%) |
| ≥65 | 40 (59.7%) |
| **Sex** | |
| Male | 61 (91.0%) |
| Female | 6 (9.0%) |
| **Ethnicity** | |
| Chinese | 50 (74.6%) |
| Malay | 8 (12.0%) |
| Other | 9 (13.4%) |
| **AFP marker levels (ng/mL)** | |
| <400 | 43 (64.2%) |
| ≥400 | 24 (35.8%) |
| **Line of systematic treatment** | |
| First | 44 (65.7%) |
| Second | 18 (26.9%) |
| Third | 5 (7.4%) |
| **Type of immunotherapy** | |
| Anti-PD-1/PD-L1 monotherapy | 41 (61.2%) |
| Anti-PD-1/PD-L1 + anti-CTLA-4 combination | 9 (13.4%) |
| Anti-PD-1/PD-L1 + others combination | 14 (20.9%) |
| Anti-CTLA-4 monotherapy | 3 (4.5%) |
| **Viral hepatitis status** | |
| HBV positive | 39 (58.2%) |
| HCV positive | 7 (10.4%) |
| Negative | 21 (31.4%) |
| **BCLC clinical staging** | |
| A | 2 (3.0%) |
| B | 7 (10.4%) |
| C | 58 (86.6%) |
| **ECOG PS scale** | |
| 0 | 46 (68.7%) |
| ≥1 | 21 (31.3%) |
| **Child-Pugh score** | |
| A5 | 40 (59.7%) |
| A6 | 21 (31.3%) |
| B7 and B8 | 6 (9.0%) |
| **Macrovascular invasion** | |
| Present | 20 (29.9%) |
| Absent | 47 (70.1%) |
| **Extra-hepatic spread** | |
| Present | 48 (71.6%) |
| Absent | 19 (28.4%) |
| **Ethnicity** | |
| Chinese | 50 (74.6%) |
| Malay | 8 (12.0%) |
| Others | 9 (13.4%) |

- Abbreviation: AFP, alpha-fetoprotein; BCLC, Barcelona Clinic Liver Cancer; CTLA-4, cytotoxic T-lymphocyte-associated protein 4; ECOG PS scale, Eastern Cooperative Oncology Group Performance Status scale; HBV, hepatitis B virus; HCV, hepatitis C virus; ICB, immune checkpoint blockade; PD-1, programmed cell death protein-1; PD-L1, programmed death-ligand 1.
- ICB-treated cohort inclusion Criteria:
  1. Histologically confirmed advanced hepatocellular carcinoma (HCC), not eligible for surgical and/or locoregional therapies; or progressive disease after surgical and /or locoregional therapies
  2. Locoregional therapy for HCC must be completed at least 4 weeks prior to the baseline scan
  3. Child-Pugh Score ≤8
  4. Eastern Cooperative Oncology Group performance status (ECOG PS) scale ≤2
- ICB-treated cohort exclusion criteria:
  1. Known fibrolamellar HCC, sarcomatoid HCC, or mixed cholangiocarcinoma and HCC
  2. Prior liver transplant
  3. Active, known, or suspected autoimmune disease

**Supplementary Table 3.** Antibodies used in immunohistochemistry.

| **Antibody** | **Dilution** | **Labeling pattern** | **Species, type** | **Company, catalog number** | **Clone** |
| --- | --- | --- | --- | --- | --- |
| LAG-3 | 1:800 | Membrane | Rabbit, monoclonal | CST, #15372 | D2G40 |
| CD8 | 1:100 | Membrane | Mouse, monoclonal | Leica, CD8-4B11-L-CE | 4B11 |
| CD68 | 1:100 | Membrane | Mouse, monoclonal | Dako, M0876 | PG-M1 |
| STAT1 | 1:10000 | Cytoplasm/ nucleus | Rabbit, monoclonal | CST, #14994 | D1K9Y |
| CD38 | 1:80 | Membrane | Mouse, monoclonal | Leica, NCL-L-CD38-290 | SPC32 (38C03) |
| PD-L1 | 1:600 | Membrane | Rabbit, monoclonal | CST, #13684S | E1L3N |

**Supplementary Table 4.** Antibodies used in flow cytometry.

| **Antibody** | **Fluorophore** | **Species, type** | **Company, catalog number** | **Clone** |
| --- | --- | --- | --- | --- |
| TCRγδ | BV480 | Mouse, monoclonal | BD Biosciences, 555717 | B1 |
| CD45 | Pacific orange | Mouse, monoclonal | Invitrogen, #MHCD4530 | HI30 |
| CD14 | BV510 | Mouse, monoclonal | BioLegend, 301841 | M5E2 |
| CD19 | BV510 | Mouse, monoclonal | BioLegend, 302241 | HIB19 |
| CD20 | BV510 | Mouse, monoclonal | BD Biosciences, 563067 | 2H7 |
| CD8a | BV570 | Mouse, monoclonal | BioLegend, 301037 | RPA-T8 |
| CD56 | BV711 | Mouse, monoclonal | BioLegend, 318335 | HCD56 |
| CD4 | BV750 | Mouse, monoclonal | BioLegend, 344643 | SK3 |
| CD3 | Alexa 532 | Mouse, monoclonal | Invitrogen, #58-0038-42 | UCHT1 |
| LAG-3 | PerCP-eFluor 710 | Mouse, monoclonal | eBioscience, #46-2239-42 | 3DS223H |
| LiveDead | Zombie NIR | NA | BioLegend, 423105 | NA |

**Supplementary Table 5.** Multivariate analysis of total LAG-3^+^, LAG-3^+^CD8^+^, CD8^+^, STAT1^+^, PD-L1^+^, CD38^+^, and CD38^+^CD68^+^ cell proportions and factors affecting progression-free survival and overall survival of ICB-treated patients (n = 67).

| **Variable** | **Progression-free Survival** | | **Overall survival** | |  |
| --- | --- | --- | --- | --- | --- |
|  | **HR (95% CI)** | ***P*-value** | **HR (95% CI)** | ***P*-value** | |
| **LAG-3^+^ cell proportion** | 0.224 (0.106-0.47) | 0.00008* | 0.307 (0.147-0.64) | 0.002* | |
| AFP | 0.59 (0.296-1.16) | 0.12 | 0.64 (0.306-1.34) | 0.23 | |
| ECOG PS scale | 0.67 (0.318-1.43) | 0.31 | 0.70 (0.258-1.91) | 0.49 | |
| Macrovascular invasion | 0.95 (0.47-1.89) | 0.88 | 0.87 (0.42-1.79) | 0.70 | |
| Child-Pugh score | 0.41 (0.153-1.10) | 0.076 | 0.44 (0.137-1.40) | 0.17 | |
| **LAG-3^+^CD8^+^ cell proportion** | 0.259 (0.123-0.55) | 0.0004* | 0.208 (0.088-0.49) | 0.0004* | |
| AFP | 0.65 (0.333-1.27) | 0.20 | 0.69 (0.327-1.44) | 0.32 | |
| ECOG PS scale | 0.74 (0.349-1.55) | 0.42 | 0.79 (0.300-2.11) | 0.64 | |
| Macrovascular invasion | 1.05 (0.54-2.06) | 0.88 | 0.96 (0.47-1.96) | 0.90 | |
| Child-Pugh score | 0.45 (0.171-1.18) | 0.11 | 0.49 (0.155-1.58) | 0.24 | |
| **CD8^+^ cell proportion** | 0.280 (0.127-0.61) | 0.002* | 0.160 (0.057-0.45) | 0.0005* | |
| AFP | 0.72 (0.374-1.37) | 0.31 | 0.71 (0.344-1.47) | 0.36 | |
| ECOG PS scale | 0.44 (0.191-1.01) | 0.053 | 0.70 (0.269-1.84) | 0.47 | |
| Macrovascular invasion | 1.20 (0.63-2.29) | 0.58 | 0.97 (0.48-1.97) | 0.93 | |
| Child-Pugh score | 0.351 (0.127-0.97) | 0.044* | 0.42 (0.138-1.28) | 0.13 | |
| **STAT1^+^ cell proportion** | 0.344 (0.136-0.87) | 0.024* | 0.276 (0.104-0.73) | 0.010* | |
| AFP | 0.48 (0.193-1.18) | 0.11 | 0.66 (0.277-1.57) | 0.35 | |
| ECOG PS scale | 0.46 (0.161-1.29) | 0.14 | 0.78 (0.212-2.83) | 0.70 | |
| Macrovascular invasion | 1.80 (0.77-4.21) | 0.17 | 1.37 (0.54-3.51) | 0.51 | |
| Child-Pugh score | 0.177 (0.044-0.71) | 0.015* | 0.096 (0.019-0.48) | 0.005* | |
| **PD-L1^+^ cell proportion** | 0.56 (0.317-0.98) | 0.044* | 0.58 (0.306-1.10) | 0.09 | |
| AFP | 0.72 (0.40-1.30) | 0.28 | 0.81 (0.42-1.53) | 0.51 | |
| ECOG PS scale | 0.81 (0.42-1.55) | 0.52 | 0.90 (0.43-1.90) | 0.79 | |
| Macrovascular invasion | 1.23 (0.67-2.27) | 0.51 | 1.04 (0.53-2.01) | 0.92 | |
| Child-Pugh score | 0.342 (0.126-0.93) | 0.036* | 0.31 (0.102-0.93) | 0.036* | |
| **CD38^+^ cell proportion** | 0.44 (0.235-0.83) | 0.011* | 0.216 (0.095-0.49) | 0.0003* | |
| AFP | 0.66 (0.373-1.16) | 0.15 | 0.72 (0.382-1.34) | 0.30 | |
| ECOG PS scale | 0.96 (0.50-1.83) | 0.89 | 1.06 (0.50-2.24) | 0.89 | |
| Macrovascular invasion | 1.12 (0.61-2.05) | 0.72 | 0.91 (0.45-1.84) | 0.80 | |
| Child-Pugh score | 0.372 (0.140-0.99) | 0.048* | 0.267 (0.088-0.81) | 0.020* | |
| **CD38^+^CD68^+^ cell proportion** | 0.51 (0.293-0.88) | 0.015* | 0.354 (0.186-0.67) | 0.002* | |
| AFP | 0.76 (0.43-1.36) | 0.36 | 0.96 (0.50-1.82) | 0.89 | |
| ECOG PS scale | 0.89 (0.47-1.67) | 0.71 | 0.98 (0.47-2.05) | 0.95 | |
| Macrovascular invasion | 1.37 (0.74-2.56) | 0.32 | 1.31 (0.66-2.61) | 0.44 | |
| Child-Pugh score | 0.43 (0.165-1.11) | 0.079 | 0.369 (0.127-1.07) | 0.07 | |

- Low is defined as AFP < 400 ng/mL, ECOG PS scale = 0, absence of macrovascular invasion, and Child-Pugh score = A5 or A6.
- High is defined as AFP ≥ 400 ng/mL, ECOG PS scale = 1, 2 or 3, presence of macrovascular invasion, and Child-Pugh score = B7 or B8.
- **P*-value <0.05 indicates statistically significance.
- Abbreviation: AFP, alpha-fetoprotein; CI, confidence interval; ECOG PS, Eastern Cooperative Oncology Group Performance Status, HR, hazard ratio; ICB, immune checkpoint blockade.

**Supplementary Table 6***.* Change in log-likelihood of models with added predictive terms for progression-free survival and overall survival of all patients (n = 67).

| **Variable** | **Progression-free survival** | | **Overall survival** | |
| --- | --- | --- | --- | --- |
|  | **∆LRχ^2^** | ***P*-value** | **∆LRχ^2^** | ***P*-value** |
| %LAG-3^+^CD8^+^ + %CD8^+^ vs %CD8^+^ | 7.9 | 0.005* | 4.9 | 0.027* |
| %CD38^+^ + %CD8^+^ vs %CD8^+^ | 4.73 | 0.030* | 11.5 | 0.0004* |
| %STAT1^+^ + %CD8^+^ vs %CD8^+^ | 5.30 | 0.021* | 4.45 | 0.035* |
| %LAG-3^+^ + %CD8^+^ vs %CD8^+^ | 9.87 | 0.002* | 4.92 | 0.027* |
| %CD38^+^CD68^+^ + %CD8^+^ vs %CD8^+^ | 9.45 | 0.002* | 6.37 | 0.012* |
| %CD38^+^ + %LAG-3^+^CD8^+^ + %CD8^+^ vs %LAG-3^+^CD8^+^ + %CD8^+^ | 2.33 | 0.13 | 9.08 | 0.026* |
| %STAT1^+^ + %CD38^+^ + %LAG-3^+^CD8^+^ + %CD8^+^ vs %CD38^+^ + %LAG-3^+^CD8^+^ + %CD8^+^ | 3.61 | 0.06 | 1.68 | 0.19 |
| %LAG-3^+^ + %CD38^+^ + %LAG-3^+^CD8^+^ + %CD8^+^ vs %CD38^+^ + %LAG-3^+^CD8^+^ + %CD8^+^ | 2.08 | 0.15 | 0.25 | 0.62 |

- % denotes the proportion of cells expressing the marker.
- As CD8^+^ cell proportion provided the best hazard ratio for OS in the multivariate analysis (Table 1), we used CD8^+^ cell proportion as the basis for comparison.
- **P*-value <0.05 indicates statistical significance, as determined with a likelihood ratio test.

**Supplementary Table 7.** Univariate and multivariate cox regression for progression-free survival of patients with viral-related (n = 46) and non-viral (n = 21) HCC treated with ICB.

| Biomarkers/ cell proportion | Viral-related HCC | | | | Non-viral HCC | | | |
| --- | --- | --- | --- | --- | --- | --- | --- | --- |
|  | Univariate analysis | | Multivariate analysis | | Univariate analysis | | Multivariate analysis | |
|  | HR (95% CI for HR) | *P*-value | HR (95% CI for HR) | *P*-value | HR (95% CI for HR) | *P*-value | HR (95% CI for HR) | *P*-value |
| LAG3 | 0.341 (0.147-0.79) | 0.012* | 0.225 (0.087-0.58) | 0.002* | 0.160 (0.045-0.563 | 0.004* | 0.124 (0.024-0.64) | 0.013* |
| LAG3^+^CD8^+^ | 0.297 (0.121-0.73) | 0.008* | 0.187 (0.068-0.51) | 0.001* | 0.268 (0.080-0.89) | 0.032* | 0.258 (0.049-1.37) | 0.11 |
| CD8 | 0.304 (0.091-1.02) | 0.053 | - | - | 0.206 (0.053-0.81) | 0.024* | 0.215 (0.052-0.90) | 0.035* |
| PD-L1 | 0.62 (0.340-1.25) | 0.2 | - | - | 0.616 (0.239-1.59) | 0.32 | - | - |
| STAT1 | 0.396 (0.089-1.77) | 0.23 | - | - | 0.201 (0.051-0.79) | 0.023* | 0.071 (0.007-0.69) | 0.023* |
| CD38 | 0.352 (0.173-0.72) | 0.004* | 0.307 (0.146-0.65) | 0.002* | 0.521 (0.181-1.50) | 0.23 | - | - |
| CD68 | 0.494 (0.221-1.10) | 0.086 | - | - | 0.430 (0.097-1.90) | 0.27 | - | - |
| CD38^+^CD68^+^ | 0.342 (0.167-0.70) | 0.003* | 0.325 (0.145-0.73) | 0.007* | 0.410 (0.130-1.29) | 0.13 | - | - |

- **P*-value <0.05 indicates statistical significance.
- Multivariate analysis was performed only in cases where the univariate analysis was significant.
- Abbreviation: CI: confidence internal; HCC, hepatocellular carcinoma; HR, hazard ratio; ICB, immune checkpoint blockade.

**Supplementary Table 8.** Multivariate Cox regression for overall survival of patients with viral-related (n = 46) and non-viral (n = 21) HCC treated with ICB.

| **Variable** | Viral-related HCC | | Non-viral HCC | |
| --- | --- | --- | --- | --- |
|  | HR (95% CI) | *P*-value | HR (95% CI) | *P*-value |
| **LAG-3^+^ cell proportion** | - | - | 0.00873 (0.00042-0.183) | 0.00227* |
| AFP | - | - | 4.018 (0.660-24.47) | 0.131 |
| ECOG PS scale | - | - | 1.601 (0.260-9.845) | 0.612 |
| Macrovascular invasion | - | - | 0.240 (0.0442-1.30) | 0.0978 |
| Child Pugh score | - | - | 2.97^-9^ (0-Inf) | 0.999 |
| **LAG-3^+^CD8^+^ cell proportion** | 0.022 (0.0027-0.184) | 0.0004* | 0.046 (0.0040-0.53) | 0.014* |
| AFP | 0.64 (0.259-1.55) | 0.32 | 2.30 (0.41-12.91) | 0.35 |
| ECOG PS scale | 0.023 (0.003-0.219) | 0.001* | 1.16 (0.236-5.74) | 0.85 |
| Macrovascular invasion | 1.85 (0.54-6.40) | 0.33 | 0.398 (0.076-2.07) | 0.27 |
| Child Pugh score | 3.20 (0.49-20.92) | 0.23 | 3.85^-9^ (0-Inf) | 0.998 |
| **CD8^+^ cell proportion** | - | - | 0.180 (0.033-0.98) | 0.049* |
| AFP | - | - | 1.15 (0.199-6.60) | 0.88 |
| ECOG PS scale | - | - | 0.69 (0.145-3.31) | 0.65 |
| Macrovascular invasion | - | - | 0.61 (0.098-3.75) | 0.59 |
| Child Pugh score | - | - | 7.84^-10^ (0-Inf) | 0.999 |
| **STAT1^+^ cell proportion** | - | - | 0.041 (0.003-0.67) | 0.025* |
| AFP | - | - | 1.25 (0.220-7.07) | 0.80 |
| ECOG PS scale | - | - | 0.317 (0.024-4.24) | 0.38 |
| Macrovascular invasion | - | - | 0.193 (0.011-3.44) | 0.26 |
| Child Pugh score | - | - | 1.13^-9^ (0-Inf) | 0.999 |
| **CD38^+^ cell proportion** | 0.158 (0.052-0.48) | 0.001* | - | - |
| AFP | 0.70 (0.321-1.53) | 0.37 | - | - |
| ECOG PS scale | 0.74 (0.308-1.79) | 0.51 | - | - |
| Macrovascular invasion | 0.81 (0.330-1.98) | 0.64 | - | - |
| Child Pugh score | 0.47 (0.130-1.72) | 0.26 | - | - |
| **CD38^+^CD68^+^ cell proportion** | 0.251 (0.109-0.56) | 0.001* | - | - |
| AFP | 0.74 (0.337-1.61) | 0.44 | - | - |
| ECOG PS scale | 0.89 (0.363-2.20) | 0.80 | - | - |
| Macrovascular invasion | 1.04 (0.45-2.40) | 0.93 | - | - |
| Child Pugh score | 0.53 (0.152-1.86) | 0.32 | - | - |

- **P*-value < 0.05 indicates statistical significance.
- Multivariate analysis was performed only in cases where the univariate analysis was significant (Table 3).
- Abbreviation: AFP, alpha-fetoprotein; CI, confidence interval; ECOG PS, Eastern Cooperative Oncology Group Perforamce Status; HCC, hepatocellular carcinoma; HR, hazard ratio; ICB, immune checkpoint blockade.

**Supplementary Table 9**. Change in the log-likelihood of models with added prognostic terms for progression-free survival and overall survival in viral-related HCC (n = 46).

| **Variable** | **Progression-free survival** | | **Overall survival** | |
| --- | --- | --- | --- | --- |
|  | **∆LRχ^2^** | ***P*-value** | **∆LRχ^2^** | ***P*-value** |
| %CD38^+^ + %LAG-3^+^CD8^+^ vs %LAG-3^+^CD8^+^ | 1.47 | 0.23 | 6.98 | 0.008* |
| %CD38^+^CD68^+^ + %CD38^+^ + %LAG-3^+^CD8^+^ vs %CD38^+^ + % LAG-3^+^CD8^+^ | 1.80 | 0.18 | 2.61 | 0.11 |
| %CD38^+^CD68^+^ + %LAG-3^+^CD8^+^ vs %LAG-3^+^CD8^+^ | 2.08 | 0.15 | 7.54 | 0.006* |
| %CD38^+^ + % CD38^+^CD68^+^ + %LAG-3^+^CD8^+^ vs %CD38^+^CD68^+^ + %LAG-3^+^CD8^+^ | 1.19 | 0.28 | 2.05 | 0.15 |

- % denotes the proportion of cells expressing the marker.
- **P*-value <0.05 indicates statistical significance.
- Abbreviation: HCC, hepatocellular carcinoma.

**Supplementary Table 10**. Change in the log-likelihood of the models with added prognostic terms for progression-free survival and overall survival in non-viral HCC (n = 21).

| **Variable** | **Progression-free survival** | | **Overall survival** | |
| --- | --- | --- | --- | --- |
|  | **∆LRχ^2^** | ***P*-value** | **∆LRχ^2^** | ***P*-value** |
| %STAT1^+^ + %LAG-3^+^ vs %LAG-3^+^ | 2.05 | 0.15 | 1.32 | 0.25 |
| %LAG-3^+^CD8^+^ + %LAG-3^+^ vs %LAG-3^+^ | 0.45 | 0.50 | 0.36 | 0.55 |
| %CD8^+^ + %LAG-3^+^ vs %LAG-3^+^ | 1.31 | 0.25 | 0.20 | 0.65 |

- % denotes the proportion of cells expressing the marker.
- Abbreviation: HCC, hepatocellular carcinoma.

**Supplementary Figure 1.** Survival outcomes of ICB-treated patients in relation to total pre-treatment CD38, CD8, STAT1, and CD38^+^CD68^+^ cell proportions. **(A-H)** Kaplan-Meier curve showing the associations between **(A and B)** high CD38^+^, **(C and D)** CD8^+^, **(E and F)** STAT1^+^, and **(G and H)** CD38^+^CD68^+^ cell proportions and better progression-free survival and overall survival after treatment with ICB. HCC, hepatocellular carcinoma; ICB, immune checkpoint blockade. **P*-value <0.05 indicates statistical significance.


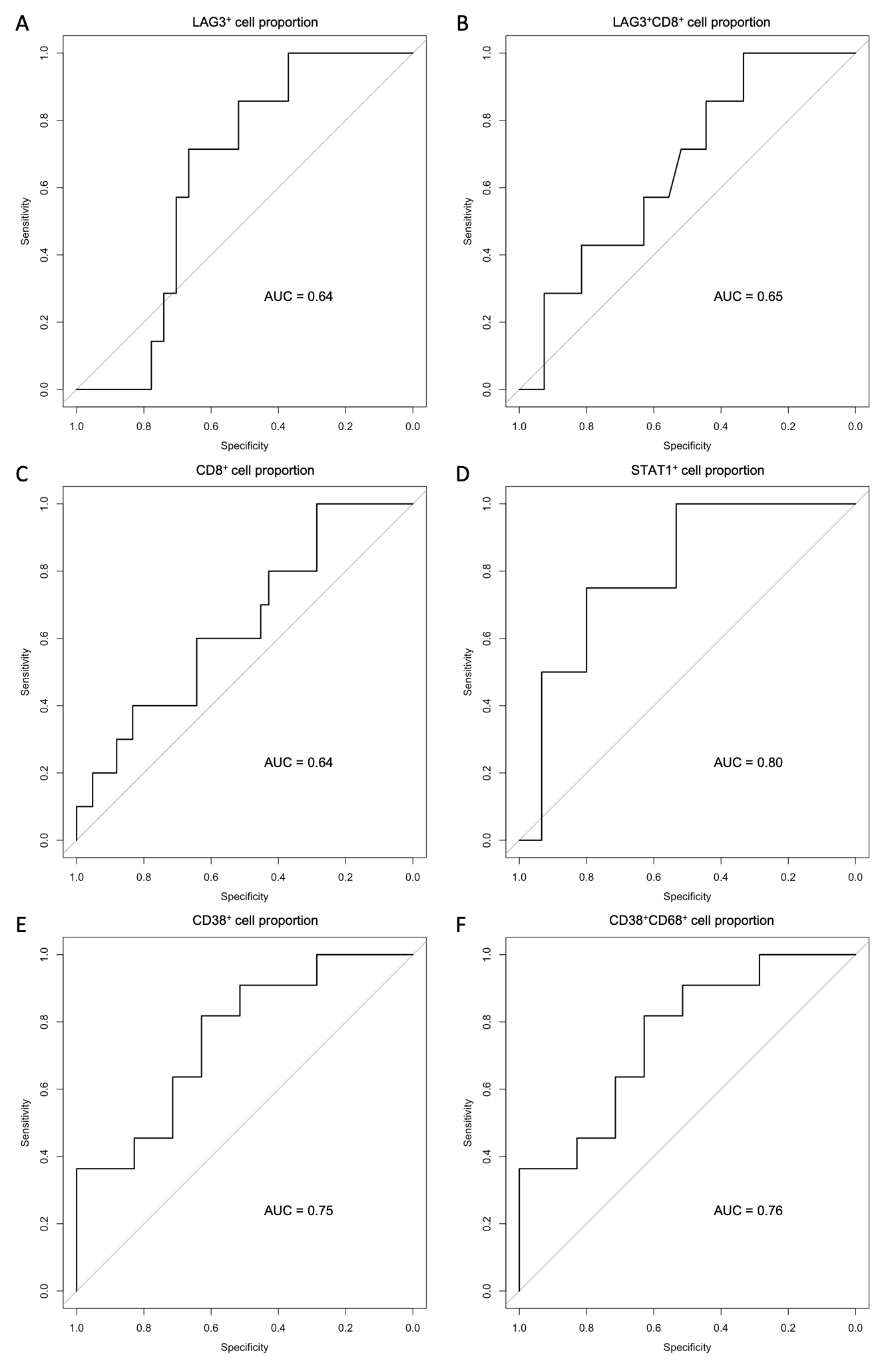


**Supplementary Figure 2**. Receiver operating characteristic curves of ICB-treated patients for **(A)** LAG3^+^ cell proportion, **(B)** LAG3^+^CD8^+^ cell proportion, **(C)** CD8^+^ cell proportion, **(D)** STAT1^+^ cell proportion, **(E)** CD38^+^ cell proportion, and **(F)** CD38^+^CD68^+^ cell proportion. AUC, area under curve.
